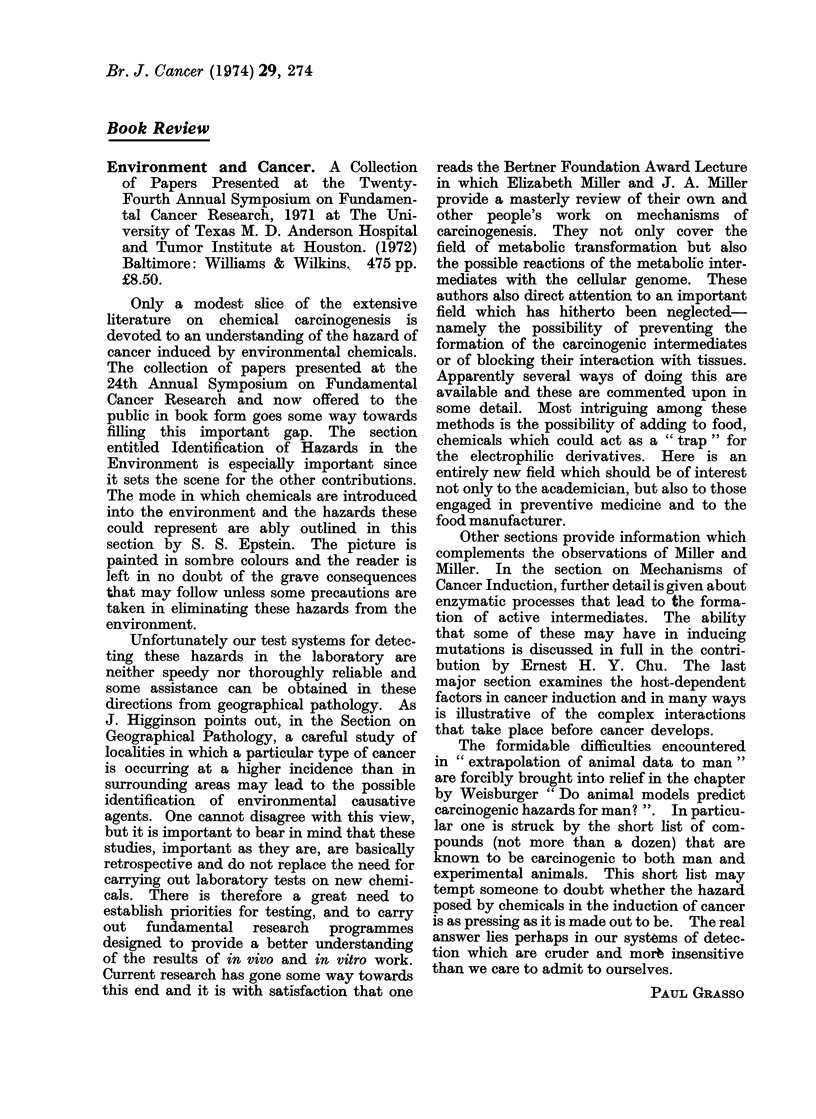# Environment and Cancer

**Published:** 1974-03

**Authors:** Paul Grasso


*Br. J. Cancer* (1974) **29**, 274

### ***Book Review***

**Environment and Cancer.** A Collection of Papers Presented at the Twenty-Fourth Annual Symposium on Fundamental Cancer Research, 1971 at The University of Texas M. D. Anderson Hospital and Tumor Institute at Houston. (1972) Baltimore: Williams & Wilkins. 475 pp. £8.50.

Only a modest slice of the extensive literature on chemical carcinogenesis is devoted to an understanding of the hazard of cancer induced by environmental chemicals. The collection of papers presented at the 24th Annual Symposium on Fundamental Cancer Research and now offered to the public in book form goes some way towards filling this important gap. The section entitled Identification of Hazards in the Environment is especially important since it sets the scene for the other contributions. The mode in which chemicals are introduced into the environment and the hazards these could represent are ably outlined in this section by S. S. Epstein. The picture is painted in sombre colours and the reader is left in no doubt of the grave consequences that may follow unless some precautions are taken in eliminating these hazards from the environment.

Unfortunately our test systems for detecting these hazards in the laboratory are neither speedy nor thoroughly reliable and some assistance can be obtained in these directions from geographical pathology. As J. Higginson points out, in the Section on Geographical Pathology, a careful study of localities in which a particular type of cancer is occurring at a higher incidence than in surrounding areas may lead to the possible identification of environmental causative agents. One cannot disagree with this view, but it is important to bear in mind that these studies, important as they are, are basically retrospective and do not replace the need for carrying out laboratory tests on new chemicals. There is therefore a great need to establish priorities for testing, and to carry out fundamental research programmes designed to provide a better understanding of the results of *in vivo* and *in vitro* work. Current research has gone some way towards this end and it is with satisfaction that one reads the Bertner Foundation Award Lecture in which Elizabeth Miller and J. A. Miller provide a masterly review of their own and other people's work on mechanisms of carcinogenesis. They not only cover the field of metabolic transformation but also the possible reactions of the metabolic intermediates with the cellular genome. These authors also direct attention to an important field which has hitherto been neglected—namely the possibility of preventing the formation of the carcinogenic intermediates or of blocking their interaction with tissues. Apparently several ways of doing this are available and these are commented upon in some detail. Most intriguing among these methods is the possibility of adding to food, chemicals which could act as a " trap " for the electrophilic derivatives. Here is an entirely new field which should be of interest not only to the academician, but also to those engaged in preventive medicine and to the food manufacturer.

Other sections provide information which complements the observations of Miller and Miller. In the section on Mechanisms of Cancer Induction, further detail is given about enzymatic processes that lead to the formation of active intermediates. The ability that some of these may have in inducing mutations is discussed in full in the contribution by Ernest H. Y. Chu. The last major section examines the host-dependent factors in cancer induction and in many ways is illustrative of the complex interactions that take place before cancer develops.

The formidable difficulties encountered in " extrapolation of animal data to man " are forcibly brought into relief in the chapter by Weisburger " Do animal models predict carcinogenic hazards for man? ". In particular one is struck by the short list of compounds (not more than a dozen) that are known to be carcinogenic to both man and experimental animals. This short list may tempt someone to doubt whether the hazard posed by chemicals in the induction of cancer is as pressing as it is made out to be. The real answer lies perhaps in our systems of detection which are cruder and more insensitive than we care to admit to ourselves.

PAUL GRASSO